# China's land carbon budget in the context of the Global Carbon Budget

**DOI:** 10.1093/nsr/nwaf073

**Published:** 2025-02-27

**Authors:** Pierre Friedlingstein, Stephen Sitch, Michael O'Sullivan

**Affiliations:** Faculty of Environment, Science and Economy, University of Exeter, UK; Laboratoire de Météorologie Dynamique, Institut Pierre-Simon Laplace, CNRS, École Normale Supérieure, Université PSL, Sorbonne Université, École Polytechnique, France; Faculty of Environment, Science and Economy, University of Exeter, UK; Faculty of Environment, Science and Economy, University of Exeter, UK

The Global Carbon Budget (GCB) produces an annual assessment of the anthropogenic perturbation of the global carbon cycle, quantifying sources and sinks of carbon dioxide (CO₂) [1]. In 2023, atmospheric CO₂ reached 420 ppm and continues to increase at ∼2.5 ppm per year. Global emissions from fossil use reached 37 billion tonnes of carbon dioxide (GtCO_2_), while global CO_2_ emissions from land-use change amounted to 3.6 GtCO_2_ per year. Over the last decade (2014–2023), the land and the ocean removed about half of the anthropogenic CO_2_ emissions, with the land and ocean sinks amounting respectively to about 12 and 10.5 GtCO_2_ per year. Both the land and the ocean sinks have been increasing over time, primarily in response to the increase in atmospheric CO₂.

**Figure 1.**
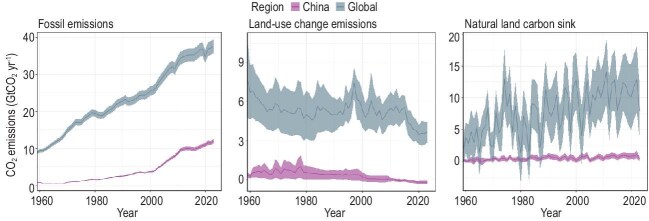
Global and China fossil CO_2_ emissions (left panel), land-use change CO₂ fluxes (middle panel) and natural land carbon sink (right panel).

Focusing now on China, fossil emissions have increased dramatically over the last decades, quadrupling over the last 30 years, reaching nearly 12 GtCO_2_ in 2023, around 30% of global fossil emissions (Fig. 1, left panel). However, China year-on-year growth in fossil emissions slowed from ∼7.5% per year during the decade 2004–2013, to <2% per year during the last decade (2014–2023), with an estimated near-zero growth for the year 2024, suggesting that a peak in Chinese CO₂ emissions might happen in the coming years.

Until 2023, GCB CO₂ emissions from land-use change (the E_LUC_ term in ref. [1]) were using Food and Agriculture Organization (FAO) country-level reporting of land cover (forest, croplands and pasture cover). For China, that led to a carbon source of about 0.25 GtCO_2_ yr^−1^. However, recent studies reported that the FAO land-cover estimates were biased over China, not properly accounting for the rapid forest expansion since the 1980s, which led to a substantial carbon sink of about 0.75 GtCO_2_ yr^−1^ [2]. The new estimates from GCB 2024 with the updated land-cover information from [2] now indicate that land-use change in China causes a net sink of ∼0.2 GtCO_2_ yr^−1^ over the 2014–2023 period (Fig. 1, middle panel). Over the same period, the GCB estimates that Chinese natural ecosystems are also acting as a carbon sink (S_LAND_ term in ref. [1]) of 0.9 ± 0.4 GtCO_2_ yr^−1^ (Fig. 1, right panel). This land sink is primarily driven by the fertilization effect, the effect of climate change being to slightly reduce the Chinese land sink by <0.1 GtCO_2_ yr^−1^. Combining the land-use induced sink (E_LUC_) and the sink in natural ecosystems (S_LAND_), we estimate China's net land CO₂ flux to be ∼1 GtCO_2_ yr^−1^.

How does the GCB estimate derived from global models compare to the new study by Xia *et al.* [3] on China's greenhouse gas budget published in this *National Science Review* Special Topic? The Xia *et al.* study uses six land carbon cycle models (IBIS, iMAPLE, LPJ-GUESS, ORCHIDEE-MICT, BEPS and TRIPLEX-GHG), the first four being also part of the GCB. The climate forcing used by Xia *et al.* (2025) is CRU-JRA merged with the ERA5 land surface reanalysis at a 0.1° × 0.1° spatial resolution, while the GCB only uses CRU-JRA at a 0.5° × 0.5° resolution. In terms of land-cover forcing, Xia *et al.* use a new land-cover-change forcing obtained by merging the Chinese Forest-Cover Dataset (CFCD) with the China Land-Cover Data (CLCD) [4,5] while GCB uses ref. [2] for China. While analysis and comparison of these land cover forcings are needed, it is beyond the scope of this short perspective.

Xia *et al.* finds a net land CO₂ flux of 1.2 ± 0.2 GtCO_2_ yr^−1^ in China, averaged over the last 10 years, reduced to 1.04 ± 0.2 GtCO_2_ yr^−1^ when accounting for carbon losses from wildfires and lateral export. Their estimate is very similar to our GCB estimate of ∼11 GtCO_2_ yr^−1^. As in the GCB, the Xia *et al.* estimate combines the effect of atmospheric CO₂, land-use change and climate change and finds that the first two are the dominant drivers of the current land sink in China. Also, the Xia *et al.* study finds a significant increase in the Chinese land carbon flux, from a small source of 0.1 GtCO_2_ yr^−1^ in the 1980s to a significant sink of 1 GtCO_2_ yr^−1^ over the last decade, again primarily driven by CO₂ fertilization and land-use change, increasing respectively by 0.54 GtCO_2_ yr^−1^ and 0.37 GtCO_2_ yr^−1^ between these two periods. In comparison, the GCB estimate indicates that the Chinese net land CO₂ flux increased from a small sink of 0.1 GtCO_2_ yr^−1^ in the 1980s to a sink of 1 GtCO_2_ yr^−1^ over the last decade, again very similar to the Xia *et al.* (2025) finding, although with a larger contribution from land-use change than CO₂ fertilization (respectively 0.6 GtCO_2_ yr^−1^ and 0.3 GtCO_2_ yr^−1^ between these two periods).

Nevertheless, both the GCB and Xia *et al.* simulate a strong increase in the Chinese net land carbon flux, from a near neutral situation in the 1980s to a significant sink over the last decade. On an area basis, this increase, over the last 40 years, is about three times larger than the global average.

More in-depth analysis of the Chinese land carbon sink, especially on the spatial patterns of the components of the land sink, will certainly come in the coming years, also making use of top-down atmospheric inversions approaches, in complement to the bottom-up approaches discussed here. Organizing a multi-model ensemble, such as TRENDY, at the country level, as already initiated by Xia *et al.*, will bring tremendous value to the community. The REgional Carbon Cycle Assessment and Process-3 (RECCAP3) activity goes in that direction [6]. Also, country-level estimates of land carbon fluxes with Dynamic Global Vegetation Models (DGVMs) will require the community to evaluate and benchmark models at the relevant spatial scale, similar to the global scale evaluation done in the GCB, in order to gain more confidence in continental to country-level assessments of the carbon budget.
